# Configuration analysis on servitisation transformation of sporting goods manufacturing enterprises: The case study of 33 listed companies of China

**DOI:** 10.1371/journal.pone.0329826

**Published:** 2025-08-05

**Authors:** Yunfei Hu, Laibing Lu, Qiuying Li

**Affiliations:** 1 Department of Sports, Henan Institute of Technology, Xinxiang, China; 2 School of Physical Education and Sport Science, Fujian Normal University, Fuzhou, China; 3 Department of Sports Rehabilitation, Hunan University of Medicine, Huaihua, China; Alcorn State University, UNITED STATES OF AMERICA

## Abstract

This study explores the multifactorial and complex causal relationships affecting the performance of sporting goods manufacturing enterprises during service transformation. A fuzzy set qualitative comparative analysis method was applied using 33 sporting goods manufacturing enterprises listed on the Shanghai and Shenzhen A-shares and the New Third Board in China. The study developed a model using the Technology-Organization-Environment(TOE) theoretical framework, to analyse the factors driving the servitisation transformation of China’s sporting goods manufacturers. The results revealed that (1) none of the seven secondary factors such as the degree of digital transformation, innovation capability, top management level, staff knowledge structure, organisational redundancy, government support, and degree of marketization individually contribute to enhancing the servitisation performance of sporting goods manufacturing enterprises in China. (2) The four grouping paths that drive the transformation of the servitisation of sporting goods manufacturing enterprises in China are divided into three types: “talent–resource driven”, “technology–organisation driven, and “technology–talent driven”. This study reveals the role of multiple factors behind the transformation of servitisation and the upgrading of sporting goods manufacturing enterprises and provides useful insights into the structural rationalisation and advanced development of China’s sports industry.

## 1. Introduction

The study of manufacturing servitisation can be traced back to Mattsson’s 1973 concept of “system selling”, i.e., “meeting a wider range of customer needs” [1], a market strategy that extends the concept of pure product selling to bundling products and services. The concept of servitizing, subsequently developed by Vandermerwe and Rada in 1988 [2], provides a strong theoretical underpinning for the development of servitized businesses in manufacturing enterprises worldwide. Servitisation brings new competitive advantages to manufacturing companies by offering a diverse range of products and services, thereby increasing their value-added. In addition, servitisation strengthens after-sales services and boosts product sales. Ultimately, these efforts have made the companies more profitable [3]. The transformation of manufacturing servitisation has become a key component of global manufacturing development. As of 2019, the number of manufacturing companies implementing service strategies reached 30% worldwide [4]. “The Guiding Opinions on Accelerating the Development of Productive Service Industry to Promote the Adjustment and Upgrading of Industrial Structure” [5], “The Special Guidelines for the Development of Service Oriented Manufacturing” [6], and “The Fourteenth Five-Year Plan” [7] stated that “Orientation towards improving quality and efficiency in the manufacturing sector promotes the deep integration of the modern service industry with the advanced manufacturing industry. The manufacturing industry has been undergone a transformation of servitisation due to increased division of labour and collaboration. Servitisation is crucial for the development of China’s manufacturing industry because of its important functions of expanding the source of value, changing value generation, and value-added methods, thus enhancing China’s global competition.

As a pillar industry for the future development of China’s national economy, in 2021, the total size of China’s sports industry reached 311.75 billion yuan, with an added value of 1224.5 billion yuan. The total scale of sporting goods and related product manufacturing was 1357.2 billion yuan, with an added value of 343.3 billion yuan, accounting for 43.5% and 28%, respectively, of the total scale and added value of the sporting industry [8]. Therefore, healthy development of the sporting goods manufacturing industry is crucial for the high-quality development of China’s sporting industry. However, in recent years, due to factors such as rising raw material costs and market tightening, the sporting goods manufacturing industry has experienced a low-end surplus, severe homogeneous competition, and restricted sales channels [9,10]. Against the background of “actively developing servitisation manufacturing and productive service industry” and the proposal of “Made in China 2025”, coupled with changes in the social and economic environment, the internal structural adjustment of the sports industry and the increasing demand for sports consumption, the transformation of the sporting goods manufacturing industry into a servitisation industry is a powerful method for adjusting the structure and enhancing the added value of sports products [11,12]. However, it is worth noting that the success of servitisation is not only a matter of “matching” between accidental factors; its causal mechanism is complex and multidimensional rather than simple and linear, and the “matching degree” between different influencing factors is crucial for transforming servitisation [13].

Existing studies have explored only the individual factors affecting the service-oriented transformation of sports manufacturing enterprises, ignoring the coordinated symbiotic relationships among multiple factors. Is the servitisation transformation of China’s sporting goods manufacturing enterprises affected by the joint impact of multiple factors? What factors have a beneficial effect on the servitisation transformation of sporting goods manufacturing enterprises in China? These questions require further investigation. Therefore, to avoid the limitations of the traditional linear model, it is important to select a suitable theoretical framework and a group perspective to explore the factors influencing the transformation of the servitisation of sporting goods manufacturing enterprises in China to support the high-quality development of the “servitisation manufacturing” of the sporting goods manufacturing industry in China.

## 2. Literature review and hypotheses

### 2.1. Literature review

#### 2.1.1. Concepts of servitisation in manufacturing.

Traditional definitions of servitisation emphasise the combination of services and products [1], and some studies consider servitisation a business model or strategy [14]. Although scholars define servitisation from different perspectives, most believe that its essence involves enterprise transformation. Specifically, studies that define servitisation as a combination of services and products describe a shift from offering products to product–service systems, whereas studies that view servitisation as a business model or strategy focus on a change in a firm’s strategic focus (from a productriented to a servitisation strategy) [15].

The former definition emphasises the outcome of servitisation (i.e., adding more services to existing products), whereas the latter emphasises a shift in strategic focus. Both definitions reflect a shift from a commodity-dominant logic to a service-dominant logic for firms. Whereas a commodity-dominant logic emphasises exchange value and treats services as a particular type of commodity, a service-dominant logic denotes a new perspective on value creation that focuses on the use value in the customer’s environment. Thus, servitisation involves rediscovering the firm’s mission, redeploying and reconfiguring organisational resources, capabilities, and structures, and renewing organisational practices, shared norms, and values [16].

At present, the manufacturing model of most Chinese sporting goods manufacturers is still Original Equipment Manufacturer(OEM) or Original Design Manufacturing(ODM), which is in the middle and lower reaches of the international sporting goods industry chain, with a focus on improving product production efficiency and product quality. However, the shift from a product-oriented to a service-oriented strategy does not meet the developmental status quo of Chinese sporting manufacturing enterprises. Therefore, this study defines servitisation as the transformation of processes by adding services to physical sports products, emphasising the outcome, and increasing the services offered to existing products.

#### 2.1.2. Research on servitisation in the sporting goods manufacturing industry.

There are two reasons for dividing manufacturing servitisation into two categories. On the one hand, manufacturing firms seek to extract additional value from services, partly to balance the increasing commoditisation of their core products and differentiate their products from those of competitors [17]. In contrast, customers’ demand for products and service products is gradually increasing [18]. Relevant studies have confirmed that the servitisation of manufacturing enterprises can significantly enhance their performance [11]. The servitisation of China’s sporting goods manufacturing enterprises has gradually attracted the attention of scholars. Early studies in China mainly focused on cooperative research and development alliances in sporting goods manufacturing enterprises, industrial agglomeration to form regional brand effects, and servitisation-production collaboration [19–22].

In recent years, with the promotion of the “Made in China 2025” strategy [23], several scholars have explored the development status of the servitisation of China’s sporting goods manufacturing industry from a variety of perspectives and proposed implementation strategies. Using the value chain theory, Li et al. [9] analysed the innovation path of the basic elements of the business model of Fujian’s sporting goods industry and explored Anta’s service innovation model in detail. Promoting the transformation of the servitisation of China’s sporting goods manufacturing industry helps build a multilevel supply system, enhances the added value of products, improves the perceived quality of customers’ sports consumption, and extends the life cycle of sports products [24]. Zhao et al. [25] argued that there are constraints, such as a lack of funds, weak independent research and development capability, and a lack of high-end composite talent, and that the industrial ecology is not yet of high quality in China’s sporting goods manufacturing industry. They proposed strategies related to concepts, scientific and technological innovation, perfecting talent projects, and optimising the business environment. On this basis, Liu [26] further proposed improving servitisation manufacturing policies, demonstrating typical servitisation manufacturing models; strengthening basic capacity building innovative internet business and service models; and strengthening the introduction of talent, training systems, and other countermeasures.

In summary, research on the servitisation of the sporting goods manufacturing industry has mostly adopted case analysis and logical thinking, which are highly subjective and require more empirical research from an objective perspective. Moreover, the literature has focused on understanding the internal logic, dilemmas, and promotional paths of the servitisation of sporting goods manufacturing enterprises. From an empirical research perspective, the specific drivers of successful servitisation implementation, as well as their interactions, have rarely been explored. The marginal contributions of this study can be summarised as follows:

(1) By analysing the real development of the sporting goods manufacturing industry in China, the technology–organisation–environment (TOE) theoretical framework is adopted to organically integrate the three influencing factors and expand the seven secondary influencing factors affecting the transformation of the servitisation of sporting goods manufacturing enterprises: degree of digital transformation, innovation ability, top management level, staff knowledge structure, organisational redundancy, governmental support, and degree of marketization. The study further conducts a fuzzy set qualitative analysis on this basis. This basis is further analysed via a fuzzy set qualitative comparison.
(2) With the help of the “group perspective”, this study uses fuzzy set qualitative comparative analysis (fsQCA) to empirically explore the concurrent synergistic effect and linkage matching mode of each conditional factor in promoting the service transformation and upgrading of China’s sporting goods manufacturing enterprises in the dimensions of technology, organisation and environment, further expanding the application of the TOE framework to explain “causal complexity”.
(3) The TOE framework has been widely used to explain the adoption of organisational technology. However, most studies have discussed the “marginal net effect” of a single condition, such as technology, organisation, and the environment, under the path of statistical regression; however, few studies have examined in detail the potential of the linkage between multiple conditions in the servitisation transformation of enterprises’ servitisation. By combining multiple conditions, this study explores the group paths affecting the servitisation transformation of sporting goods manufacturing enterprises, which helps reveal the “black box” of this transformation and enriches and expands the related theoretical research in the Chinese context.

### 2.2. Theoretical framework construction and hypotheses

#### 2.2.1. TOE framework.

It is difficult to explain the success of servitisation in manufacturing firms by investigating the factors in isolation, but it is more accurate to understand this success from the perspective of balanced configurations [27]. The same is true for sporting goods manufacturing enterprises, for which a multidimensional complex path provides a more accurate description of reality than a single influencing factor. The development of a servitisation business should consider the characteristics of the enterprise’s internal technical structure and organisational resources and clarify the coupling effect brought about by external environmental factors, such as the government and the market [28].

The TOE framework was proposed in 1990 by Tornatizky et al. [29] and is useful for explaining the causes of complex social phenomena and extracting influencing factors [30]. The TOE framework argues that new technologies are subject to a combination of technological, organisational, and environmental factors in the application process. Among these factors, technological context factors refer to internal and external technologies relevant to the organisation and are available for adoption. Organisational contextual factors refer to the descriptive characteristics of a company, its resources, and communication processes among its employees. Environmental context factors include market elements, competitors, and regulatory environment [29]. The TOE framework has been used to analyse the servitisation transformation of manufacturing enterprises in China [31–33], and has good interpretative validity.

The servitisation of sporting goods manufacturing can be regarded as an “extension” of manufacturing technology. The profitability of a service product is influenced by numerous factors, including customer interest in service products [34], static and dynamic organisational elements [35], and the business environment [36], which are also in line with the analytical dimensions of the TOE framework and reflect its strong advantages of the TOE framework in explaining the servitisation of the sporting goods manufacturing industry. This framework has strong advantages in explaining the servitisation of sporting goods manufacturing. Therefore, drawing on the existing research results, this study adopts the TOE model as the theoretical framework, combines the practical scenario of servitisation in China’s sporting goods manufacturing industry, and further identifies the conditional variables affecting the industry’s servitisation transformation in terms of the three dimensions of technology, organisation, and environment.

The selection of influencing factors for each dimension is based on a classical knowledge production function. The knowledge production function was first proposed by Griliches and expanded and improved by Jaffe [37] in 1989. It has been widely used in research related to the factors influencing knowledge production and technological innovation. Its functional relationship is expressed in the form of a Cobb–‒Douglas production function as follows:


$$Y_{i,t}=K_{i,t}^{\alpha }L_{i,t}^{\beta }Z_{i,t}^{\gamma }\varepsilon_{i,t}$$ (1)

where *Y* is the service-oriented transformation performance of the sporting goods manufacturing industry; *K* is the capital input in the process of improving service-oriented transformation performance; *L* is the labour input in the process of improving service-oriented performance; *Z* represents the other variables; and i and t denote the object and period of the study, respectively. *α*, *β,* and *γ* are the constant regression parameters to be estimated, and *ε* is the random disturbance term, which denotes the unobserved influencing factors. Thus far, based on the research purpose of this study, combined with the TOE framework, capital input is measured by the degree of digital transformation, innovation ability, and organisational redundancy of Chinese sporting goods manufacturing enterprises. Labour input is measured by the level of top management and the knowledge structure of employees, while considering the influence of other factors such as environmental factors and further adding indicators such as the strength of government subsidies and marketization. Finally, the Cobb–Douglas production function and TOE framework are integrated to formulate hypotheses about the relationships between the influencing factors and the performance of servitisation transformation.

#### 2.2.2. Hypotheses.

(1) Technical dimension factors: Technological upgrading is an important foundation for the servitisation transformation of enterprises, which is manifested in two main aspects: the use of digital technology and the upgrading of product technology. In recent years, the rapid development of digital technology has made ubiquitous customer communication possible, leading to the acquisition, storage, and analysis of big data that are widely used in personalised services and deeper customer relationships [38]. The digital economy has produced profound changes in the traditional manufacturing industry, not only by changing the production mode and improving work efficiency but also by reshaping the business model and ecosystem. In the service production of enterprises, the real-time analysis of customer behaviour, demand preferences, and other laws using information provided by digital technology can rapidly capture the potential needs of customers and flexibly adjust the service scheme precisely and efficiently [39].

In summary, digital technology provides accurate decision-making support; reduces unnecessary waste of human, material, and financial resources; and thus promotes the synergistic development of servitisation consciousness, capability, and performance of enterprises. Moreover, technological innovation not only enhances the professional degree of the modern service industry but also provides support and guarantees for the transformation of servitisation in traditional manufacturing industries [40]. The process of servitising manufacturing enterprises cannot be separated from investment in product innovation resources, which is an important way to ensure the high-quality output and sales of servitisation products. The research and development of new products can influence consumers’ expectations of the benefits of products and improve the quality of service products while also shaping the image of high-quality products. Products with high technological content also increase the intensity of market competition and improve enterprise servitisation performance [41].

In addition, the impact of innovation capability on firms’ servitisation performance can also be regarded as the enhancement of the quality of service factors on the efficiency of their utilisation; technological innovation capability can achieve technological leapfrogging by overcoming key technological bottlenecks, which can further contribute to the transformation and upgrading of servitisation in manufacturing firms. In addition, factors such as dynamic capabilities (coordination and integration capabilities, learning capabilities, and reconfiguration capabilities) are key drivers of servitisation [42]. Accordingly, the following research hypothesis is proposed.

H1: Conditional configurations involving digital technology development and product upgrading are necessary conditions for the servitisation transformation of China’s sporting goods manufacturing enterprises.

(2) Organisational dimension factors: Organisational management provides an important growth space for the servitisation transformation of enterprises, which is manifested mainly in three aspects: senior management level, staff knowledge structure, and organisational redundancy. According to the theory of human capital, the difference in the growth rate between inputs and outputs is partly due to the technological progress brought about by human capital, which in turn is closely related to the enterprise’s senior management level and staff knowledge structure [43]. To increase the market competitiveness of products, the complexity of product and technology matching in sporting goods manufacturing enterprises is increasing, and servitisation can significantly increase the value of products. To achieve this goal, the enterprise’s senior management takes the initiative to develop the service market through the formulation of an enterprise development strategy and simultaneously strengthens the cultivation of enterprise servitisation employees [44]. Senior managers can also identify the typical characteristics and development trends of manufacturing servitisation, such as economies of scale, and formulate corresponding development strategies for the future transformation and development of the enterprise so that the enterprise can take the initiative in fierce market competition [45]. In summary, the importance and management level of service at the senior management level of an enterprise affects the servitisation level of the manufacturing enterprise.

In addition, the staff knowledge structure has a two-fold influence on improving the level of servitisation in manufacturing enterprises. On the one hand, with the continuous improvement of the technology of manufacturing products and the continuous refinement of service products, many knowledge-based employees are needed to improve production efficiency to meet customer demand through the division of labour and cooperation of the employee team [46]; on the other hand, as knowledge-based employees can provide professional and knowledge-intensive services, an accurate match between the manufacturing industry’s products and the supply of service demand can be achieved [47,48]. Organisational redundancy reflects the degree of excess resources in a firm. This is defined as the gap between a firm’s existing resources and those required to maintain production operations, or unused idle resources [49]. The service business of manufacturing enterprises differs from the traditional manufacturing business, which requires a large and new injection of capital strongly supported by the redundant resources of the enterprise. Organisational redundancy, while providing resources for service innovation in manufacturing firms, can also promote the transformation of servitisation and upgrading of manufacturing firms by reducing resistance to resource rigidity.

H2: Conditional configurations involving top management, staff knowledge structure, and organisational redundancy are necessary conditions for the servitisation transformation of China’s sporting goods manufacturing enterprises.

(3) Environmental dimension factors: Changes in the external environment are an important opportunity for the transformation of the servitisation of enterprises, which manifests mainly through government support and the degree of marketization. When a region has a high degree of marketization, laws and regulations are relatively complete, and the government’s policy formulation and implementation are more established. This can increase the confidence of manufacturing enterprises in the region in conducting various service businesses and provide a solid policy and market foundation for the transformation of enterprise servitisation [50].

Moreover, regions with a higher degree of servitisation have greater factor mobility and a more efficient allocation of resources, which can promote improvements in the production efficiency of manufacturing enterprises and the transformation and upgrading of industries [51]. The implementation of servitisation in the manufacturing industry implies that the value chain of traditional manufacturing enterprises extends to both ends, which requires more human capital, R&D and design, information technology, marketing management, after-sales maintenance, and other high-quality service elements [52],therefore, strong financial support is essential. Government subsidies can increase cash flows for manufacturing enterprises, enabling them to conduct more highly targeted investments [53]. Government financial subsidies also send positive signals to the market, making it easier for enterprises to obtain external financing in the financial market [54]. Therefore, manufacturing enterprises with strong government subsidies and sufficient government financial support can fully expand the scale of their enterprise servitisation strategies and increase the number of servitisation businesses to enhance servitisation transformation performance.

H3: Conditional configurations involving the strength of government support and the degree of marketization are necessary conditions for the servitisation transformation of China’s sporting goods manufacturing enterprises.

## 3. Research design

### 3.1. Research methodology

Fuzzy set qualitative comparative analysis (fsQCA) is an ensemble theory approach for identifying configurations of conditions that are necessary or sufficient for an outcome to occur through the use of Boolean algebra and logical minimisation rules and is suitable for exploring the cumulative effects of different constituent factors [55]. According to the configuration theory, different configurations of causal factors can produce the same outcome. FsQCA can integrate structural, strategic, and environmental factors and is suitable for capturing complex causal relationships. Compared with deterministic set qualitative comparative analysis (mvQCA) and multivalued set qualitative comparative analysis (csQCA), fsQCA can convert antecedent conditions and outcomes into any number between 0 and 1, which is suitable not only for dealing with category problems, but also for dealing with degree changes and partial affiliation problems. In conjunction with the selected topic of this study, the fsQCA approach helps decision-makers in sporting goods manufacturing companies choose from different relationship governance strategies to select a reasonable path to improve the performance of service-based transformation. This study is analysed using fsQCA 3.0 software.

### 3.2. Sample selection and data source

This study considers sporting goods manufacturing enterprises listed on the Shanghai and Shenzhen A-shares and New Third Board. The selection criteria are as follows: (1) the selected enterprises comply with the relevant provisions of the national statistical classification of the sporting goods industry and conduct the service business during the sample years; (2) the enterprise’s business scope includes the sporting goods manufacturing business, which owns its brand and accounts for more than 50% of the company’s overall revenue; (3) there are no major financial errors in the enterprise’s annual report; and (4) the sample years of enterprises are excluded from ST, SST, S*ST, *ST, or PT. The conditional variable selects the enterprise financial data in 2020, and the impact of related factors on enterprise service needs a process, so the result variable selects the enterprise financial data in 2021. The financial indicators are mainly taken from the Cathay Pacific database, the Juchao Information Network, and enterprise annual report data. Finally, 33 listed sporting goods manufacturing enterprises were screened; their basic information is shown in Table 1.

**Table 1:** Basic information about the study sample.

Security Code	Enterprise Name	Registered Address	Security Code	Enterprise Name	Registered Address	Security Code	Enterprise Name	Registered Address
002780	Sanfo Outdoor	Beijing	603129	Chunfeng Power	Hangzhou	603908	Comefly	Ningbo
300005	Toread	Beijing	603558	Jasan Group	Hangzhou	002489	Zhejiang Yongqiang	Taizhou
838464	Carving Ski	Beijing	430759	Cronus	Xiamen	002870	Senssun	Zhongshan
833151	Tongfang Health	Beijing	603555	Guirenniao	Xiamen	300526	China Dive	Huizhou
833429	Competitor	Beijing	837720	Youli Sports	Nantong	002899	Impulse	Qingdao
871594	Cnsg Holdings	Beijing	836210	Sumar Marine	Nantong	833603	Aosen Garment	Baoding
002395	Double Elephant	Wuxi	830877	Kanglai Sports	Taizhou	300651	Jinling Sports	Suzhou
837226	Lianchuang Artificial Lawn	Wuxi	871721	Source One Outdoor	Taizhou	831326	Sanlida	Jiaozuo
873009	Sifang Swimming	Wuxi	002832	Biemlfdlkk	Guangzhou	834261	Inov	Zibo
600679	Shanghai Phoenix	Shanghai	002105	HL Corp	Shenzhen	870749	Jianhua Zhongxing	Jining
002486	Challenge	Shanghai	833649	Beaume Outdoor	Nanjing	832875	Fushide	Wuhu

Note: The above corporate information was obtained from the official websites of the Shanghai Stock Exchange and Shenzhen Stock Exchange.

### 3.3. Variable measurement and calibration

#### 3.3.1. Variable measurement.

(1) Outcome variable measurement

Measuring the performance of a firm’s transformation of servitisation needs to be considered from multiple perspectives and should include data on firm development at both the financial and non-financial levels [56]. Financial performance reflects the extent to which a company achieves its economic goal. For example, Xiao’s [57] approach measures the financial performance of the transformation of servitisation by adopting the “ratio of service business revenue to total revenue”, in which the service business revenue is the sum of the revenues of the eight categories of productive service items related to the main business. In addition, existing research suggests that the labour force is shifting from manufacturing to services [58], and considering the dependence of the service business, nonproduction employees play an important role in the service-oriented transformation of manufacturing companies. Non-production employees include managers, engineers, technicians, service personnel, and others other than direct production staff whose job tasks involve decision-making and planning, which are seen as enduring and not responsive to immediate market demand [59]. The long-term business viability of a firm is seen as deriving more fundamentally from nonproduction employees, who are focused more on product development, innovation, consumer demand analysis, “information handling” and the development of new technologies and production processes [60]. Accordingly, the “proportion of non-production employees to all employees” is used as a proxy variable for the staffing structure of the service business and for measuring the non-financial performance of firms’ servitisation transformation. Notably, personnel unrelated to service operations, such as financial and “administrative personnel”, were excluded in the selection of non-production employees. Finally, the entropy method is used to calculate the total score for the service transformation performance of sporting goods manufacturing enterprises in China.

(2) Conditional variable measurements

Combining the research purpose of this study with the reality of sporting goods manufacturing enterprises in China, the degree of enterprise digitisation (*DIG*) and innovation capability (*INN*) are used to measure the technological dimensional factors; senior management level (*MAN*), staff knowledge structure (*STA*), and organisational redundancy (*OS*) are selected to measure the organisational dimensional factors; and the strength of government subsidies (*SUB*) and the degree of marketization (**M*AR*) are selected to measure the environmental dimensional factors (Table 2).

**Table 2:** Variable selection and measurement.

Variable type	Measurement dimension	Variable name	Variable symbol	Measurement method
Outcome variable		Performance of transformation of servitisation	*SV*	Calculate “service business revenue/total business revenue” and “number of nonproduction employees/total number of employees” separately, and then adopt the entropy method to calculate the total score.
Conditional variable	Technical dimension	Degree of digital transformation	*DIG*	Constructing a thesaurus for digital transformation and counting the frequency of digitisation-related words in annual reports of enterprises
		Innovation ability	*INN*	R&D expenditure/operating income
	Organisational dimension	Senior management level	*MAN*	Operating income/average total assets
		Staff knowledge structure	*STA*	Percentage of employees with a specialised degree or above
		Organisational redundancy	*OS*	(Selling expenses + Administrative expenses + Financial expenses)/Sales revenue
	Environmental dimension	Strength of government subsidies	*SUB*	Amount of government grants published in the annual reports of enterprises
		Marketization process	*MAR*	“FanGang index” of the city where the enterprise is located

1) The degree of digital transformation was constructed by referring to the **Digital China Development* Report (2021)* released by the State internet Information Office as well as authoritative policy documents, such as the Government Work Report, in recent years to construct a thesaurus of 116 digitalisation-related vocabularies, and at the same time, drawing on the relevant authoritative literature [61], using the frequency number of digitised keywords in the annual report as an indicator of the degree of digitisation; 2) innovation capability can reflect the vitality of various innovative activities of enterprises, and is expressed by the widely used R&D investment density (R&D expenditure/operating revenue) [62]; 3) the management level of top management is expressed by the total asset turnover ratio [63]; 4) staff knowledge structure is expressed by the proportion of tertiary educated employees to total employees [64]; 5) organisational redundancy is measured using the enterprise sales period expense ratio, i.e., the sum of the enterprise’s selling, administrative and financial expenses as a proportion of sales revenue [65]; 6) the strength of government subsidies is measured by the number of government subsidies disclosed(million) in the notes of the enterprise’s annual report [66]; and 7) marketization is expressed using the marketization process index proposed by Fan [67], which, through a set of complete and objective indicator systems, can measure the degree of marketization of the enterprise’s location city and achieve a comprehensive score. The measurement includes five major aspects: the relationship between the government and the market, the development of the non-state economy, the development of the product market, the development of the factor market, the development of intermediary organisations in the market, and the legal and institutional environment.

#### 3.3.2. Variable calibration.

Calibration is the process of assigning set affiliations to cases. This study uses a direct calibration method to convert the raw data into fuzzy set affiliation scores, and the calibrated set affiliation is between 0 and 1. According to related studies [68,69], the 95%, 50% and 5% quantile values of the outcome variable and the condition variable are chosen as the “fully affiliated”, “intersection” and “fully unaffiliated” values, respectively. The calibration results are presented in Table 3.

**Table 3:** Calibration anchors for each variable.

Variant	Full affiliation point	Crossover point	Totally unaffiliated point
Performance of transformation of servitisation	0.761	0.091	0.018
Degree of digital transformation	4.454	2.398	0.693
Innovation ability	6.471	3.634	1.076
Senior management level	2.182	0.775	0.194
Staff knowledge structure	0.573	0.130	0.021
Organisational redundancy	0.557	0.169	0.041
Strength of government subsidies	28.713	2.834	0.103
Marketization process	18.617	14.846	11.163

## 4. Data analysis and empirical results

### 4.1. Necessity analysis

Necessity analysis is an important procedure in fsQCA that tests whether a single condition is necessary for the occurrence of an outcome. Referring to common treatments in existing studies, a single condition is necessary for the outcome when the consistency level is greater than 0.9 [69]. The results of the necessary condition analysis are presented in Table 4, where the consistency level of all the condition variables is less than 0.9. Therefore, there is no necessary condition that affects the high and low levels of servitisation transformation of sporting goods manufacturing enterprises. Moreover, most of the consistency levels of the high- and low-level transformations of servitisation are less than 0. 75, which is at a lower level, indicating that the conditions that cause the transformation of the servitisation of sporting goods manufacturing enterprises are the result of the synergistic effect of multiple conditions, such as technology, organisation, and the environment.

**Table 4:** Results of the necessary conditions analysis.

Conditional variable	High-level of servitisation		Low-level of servitisation	
	Degree of consistency	Degree of coverage	Degree of consistency	Degree of coverage
High degree of digital transformation	0.669	0.619	0.614	0.658
Low degree of digital transformation	0.627	0.585	0.642	0.691
High innovation ability	0.673	0.613	0.688	0.723
Low innovative ability	0.695	0.659	0.632	0.690
High management level	0.631	0.640	0.656	0.768
Low innovative ability	0.771	0.661	0.692	0.684
High staff knowledge structure	0.774	0.773	0.510	0.587
Low staff knowledge structure	0.586	0.509	0.803	0.804
High organisational redundancy	0.783	0.734	0.578	0.625
Low organisational redundancy	0.601	0.553	0.754	0.800
High strength of government subsidies	0.609	0.640	0.555	0.672
Low strength of government subsidies	0.688	0.573	0.702	0.674
High marketization process	0.613	0.577	0.692	0.751
Low marketization process	0.736	0.674	0.611	0.645

### 4.2. Group state analysis of the transformation of the servitisation of sporting goods manufacturing enterprises

Unlike the necessary condition analysis, conditional group state analysis is based mainly on the set theory perspective to explore the level of sufficiency of different group states, triggering results composed of multiple conditions. The consistency threshold is an important criterion for group state sufficiency analysis. According to Schneider [70], this paper sets the adequacy consistency level threshold to 0.80. In addition, the number of cases included in the grouping is the screening criterion for a particular grouping to enter into the Boolean minimisation calculation, and the frequency threshold must be determined based on the sample size.

Therefore, in conjunction with the sample size of this study and with reference to Ding [71], the frequency count threshold was set to 1, whereas the PRI consistency level threshold was set to 0.6 for the normalisation run, resulting in complex, intermediate and parsimonious solutions. By performing Boolean algebraic operations on the intermediate and parsimonious solutions, grouping results were obtained, as shown in Table 5.

**Table 5:** Results of the transformation of servitisation grouping analysis.

Table 5 shows that four configurations can lead to the high-level transformation of servitisation and the development results of sporting goods manufacturing enterprises, which can be classified into three types of configurations according to the combination of their core conditions, i.e., “talent–resource driven” (corresponding to configurations A1 and A2), “technology–organisation driven” (corresponding to configuration B) and “technology–talent driven” (corresponding to configuration C). The consistency index of each configuration ranges from 0.900 to 0.952, which meets the criteria proposed by Woodside [72]. The overall consistency is 0.921, indicating that the degree of explanation of the three types of grouping paths for the high-level transformation of the servitisation of sporting goods manufacturing enterprises in China is 92.1%. The overall coverage is 0.607, indicating that the results of the empirical analyses in this study can eventually cover 60.7% of the case scenarios. The consistency of all the group paths is greater than 0.8, indicating that there is a good subset relationship between these group paths and the high-level transformation of the servitisation of sporting goods manufacturing enterprises; that is, the antecedent conditions have good explanatory power for the outcome variables, and this result also confirms H1–H3.

**Fig 1:**
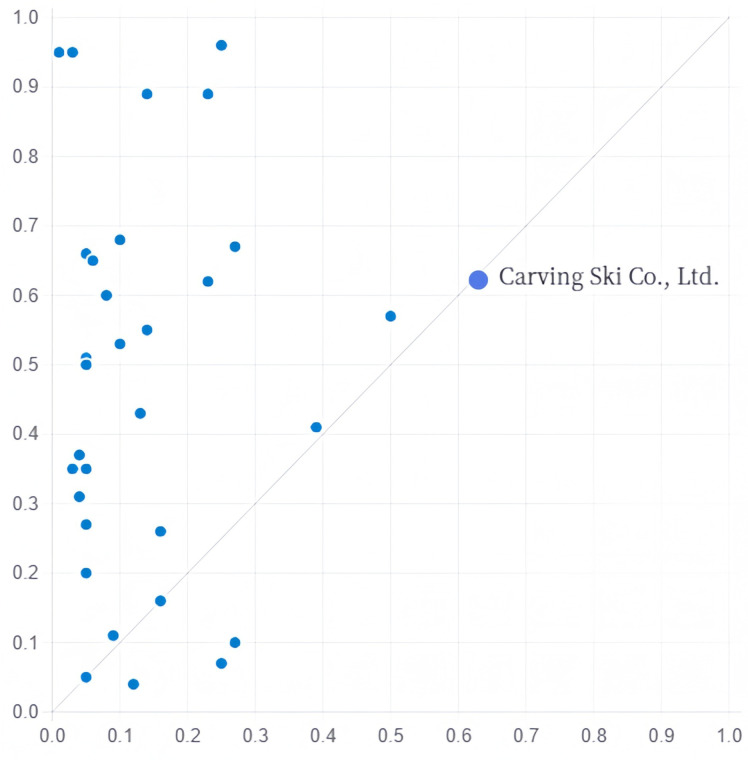
Explanation case of configuration A1. (Note: In Fig 1, the x- and y-axes represent the fuzzy set membership values of the case on the condition variable and the outcome variable, respectively. The closer the corresponding value of the case is to 1, the higher the conformity of the case in that configuration. The following picture is the same).

(1) Talent–resource driven. This grouping path consists of subconstructs A1 and A2, where A1 has a likelihood of 0.909, explaining 28.7% of the transformation of servitisation cases of sports manufacturing enterprises, of which 7.6% can be explained by this path only, with the highest unique coverage of all grouping paths. The presence of an staff knowledge structure, organisational redundancy, and absence of government subsidies are the core conditions in this path for sports manufacturing firms. This construct shows that when the proportion of employees with high education levels and organisational redundancy in sports manufacturing firms is high, even if government support is lacking, it can promote the servitisation transformation of sports manufacturing firms, as in the case of Carving Ski Co., Ltd. (Fig 1).

**Fig 2:**
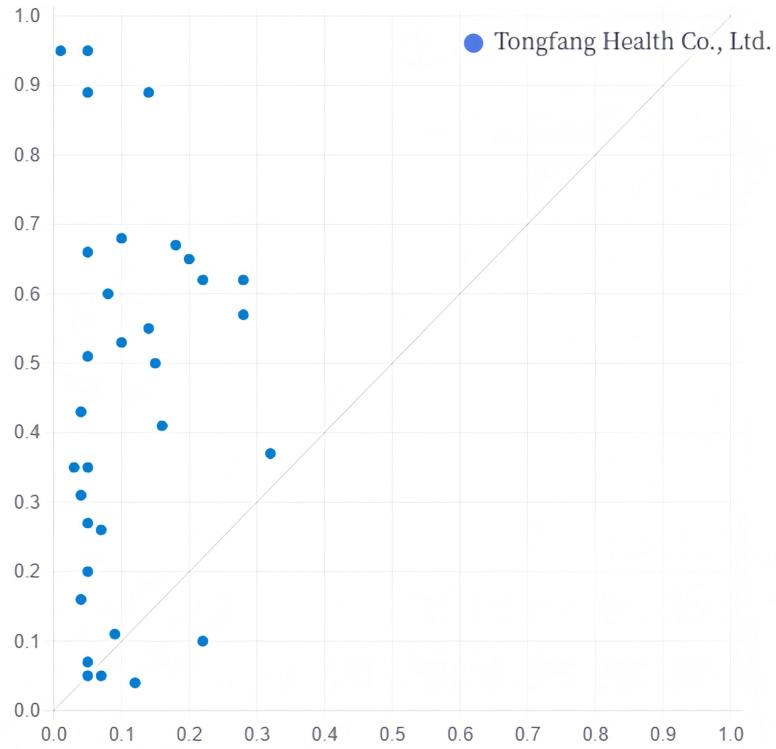
Explanation case of configuration A2.

Subgroup configuration A2 has a likelihood of 0.947, explaining 25.6% of the servitisation transformation cases of sports manufacturing enterprises, of which 4.4% can be explained only by this path. The core conditions in this pathway are consistent with those in A1, with only a few adjusted non-core conditions, and the representative case is Tongfang Health Co., Ltd. (Fig 2).

Organisational redundancy reflects the flexibility attributes associated with collective action in business organisations and can support collective action by enhancing the reliability of subsystems and enabling real-time collaboration between different subsystems [73]. Organisational redundancy can also provide critical information for real-time product transformation and upgrading to enhance the reliability of product development and multilocation coordination. It has a dual impact on service innovation and service transformation in sporting goods manufacturing enterprises: organisational redundancy can provide resources for service innovation and reduce the resistance of enterprise resource rigidity; while it can also promote cross-boundary search activities for external knowledge, which can also help reduce the resistance of enterprise management rigidity, which can indirectly promote the service innovation of manufacturing enterprises [74]. Moreover, owing to the high technical content of servitisation products, employees are required to have a strong professional background and efficient practice ability, which contributes to the construction of the enterprise’s servitisation business platform and the optimisation and upgrading of servitisation product technology. The service of a product is the result of the cooperation of employees with different professional knowledge backgrounds. organisational redundancy can also effectively integrate creative abilities among employees, enhance the transfer and integration of related technologies, and make collective action in the organisation possible [75]. When the environment changes, organisations may not be able to rely on established rules and procedures to manage the interdependencies between different organisational functions and members. Instead, they need to use new information and knowledge to adapt to new opportunities [76]. Therefore, the redundancy of organisational information among members facilitates real-time communication and coordination, thus contributing to new product development in dynamic environments.

Consider Cabin Ski Co. Ltd. as an example. Its headquarters is located in Beijing, and it is a comprehensive enterprise that focuses on services to the ski industry; selling large-scale ski equipment as their main products. The company provides professional and comprehensive service solutions to customers at different developmental stages and with different business needs. The company makes every effort to develop new markets while improving internal governance, optimising the organisational structure, further clarifying job duties and obligations, strengthening team building, and improving personnel assessment and incentive mechanisms. Optimising service capacity increases the customer base. Moreover, in recent years, with the advantage of location, the company has attracted a large number of highly educated and skilled employees. In 2021, the company’s service business revenue increased substantially compared to the previous year, while the gross profit margin reached 160.28%, successfully completing the transformation and upgrading of the service business.

**Fig 3:**
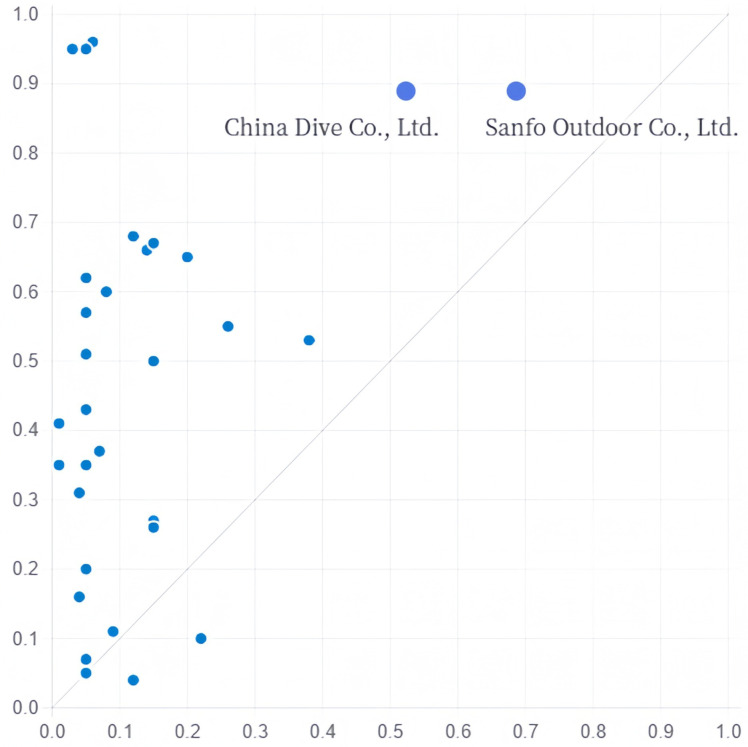
Explanation case of configuration B.

(2) Technology–Organisation Driven. This driving path encompasses Group B. The likelihood of their existence is 0. 952, which is the highest among all grouping paths and accounts for 26.3% of the service transformation cases, of which 6.9% can be explained by this path only. Digital transformation, the existence of an staff knowledge structure, the existence of organisational redundancy, the absence of innovation capability, the absence of a top management level, the absence of government subsidies, and the absence of marketability are the core conditions in this path. In other words, when the degree of digital transformation, proportion of highly educated employees, and organisational redundancy are high in sporting goods manufacturing enterprises, even if the innovation ability and top management level are not high and government and market support are out of place, it can still contribute to improving the service transformation performance of sporting goods manufacturing enterprises, as exemplified by the cases of China Dive Co., Ltd. and Sanfo Outdoor Co., Ltd. (Fig 3).

In implementing the enterprise service process, in addition to the advantages of technical talent and potential resource support, which can be buffered by the enterprise, digital technology can respond to the instability of technological innovation and market changes through the stability of digital flow, improve the efficiency of resource allocation, and enhance the profitability of the servitisation business [77]. The impact of the new value concept of digital technology and the traditional business philosophy will bring corresponding changes to the enterprise’s organisational structure and business processes, inducing the enterprise to make adaptive changes in the production, sales, and after-sales links and comprehensively upgrade the execution of all process links, including the transformation of the product service. However, the digital economy reduces the costs of searching, copying, transport, traceability, and authentication for enterprises and significantly improves supply chain coordination and the ability to control production risk.

For example, China Dive Co., Ltd. is a member of the diving and underwater salvage equipment manufacturing industry. The main business scope is research and development, production, and sales of protective diving gear. The services provided include big data internet services, diving technology training, and travel assistance services. The company has vigorously developed big data internet technology in recent years, and while the revenue from the service business has been increasing annually, the proportion of the company’s big data service revenue to the overall service revenue has also shown rapid growth. Moreover, by constructing an intelligent manufacturing system, efficient operation can be achieved, which can better meet the customised needs and quality requirements of high-end customers, while reducing manpower costs, improving production efficiency, and overcoming existing capacity bottlenecks.

**Fig 4:**
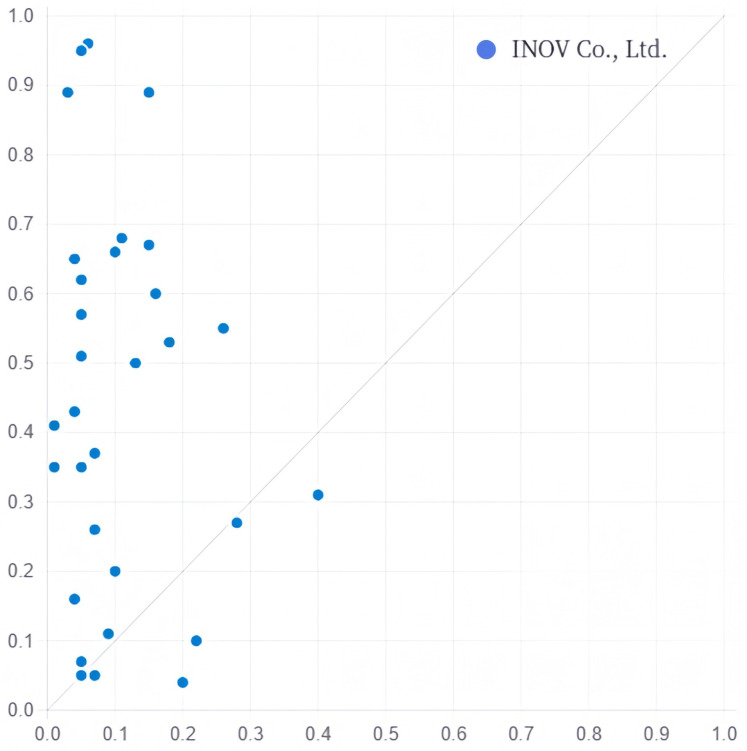
Explanation case of configuration C.

(3) Technology–Talent Driven. This driving path includes grouping C. The probability of existence is 0.9, explaining 23.6% of the transformation of servitisation cases, of which only 6.2% can be explained by this path. The existence of digital transformation, the existence of an staff knowledge structure, and the absence of a marketization process are the core conditions in this path. That is, when the degree of digital transformation and the proportion of highly educated employees are high in a sports manufacturing company, it can contribute to the transformation of servitisation of the sports manufacturing company, even if the innovation capacity is low and the marketization process is slow. A typical case is INOV Co., Ltd. (Fig 4).

Upgrading industrial structure is accompanied by an improvement in the quality of human capital. The construction of a strong manufacturing country has given rise to qualitative and quantitative demand for skilled personnel, which should not only focus on the quantity of skilled personnel, but also pay more attention to the talent dividends produced by improving the quality of skilled personnel. The popularity of the new generation of information technology in the industrial field has promoted changes in manufacturing methods but also promoted the optimisation of the knowledge structure of talent, and “digital chain upgrading” and “optimisation of the talent chain” have contributed to the dynamic upgrading of the competitive advantage of the manufacturing industry. Currently, the digital talent training of sports enterprises in China has unclear standards and digital transformation strategies that are not conducive for cultivating digital talent in sports enterprises [78]. To improve the service capacity of sporting goods manufacturing enterprises, we should rely on combining a “knowledge factor set” and a “traditional factor set” with different levels of talent at the core to achieve industrial advancement through diversified industrial upgrading activities.

INOV Co., Ltd., is China’s leading sports venue facility engineering enterprise. The company adheres to customer demand, constantly strengthens the construction of digital technology, improves the conditions of production automation and lean production management, improves the procurement system, and continues to optimise product costs. The sales model for big data internet analysis supports the accurate positioning of customer groups. Simultaneously, the company assigns great importance to the introduction and cultivation of high-level talent. In 2020, 59.6% of the R&D personnel held master’s degrees and doctorates through professional skills training, management training, job rotation and exercise, external learning, and other modes of continuous improvement in the professional competence and comprehensive level of employees, the pursuit of employees, and the enterprise’s common growth. The development path of “digital technology construction + talent cultivation” enabled the company to increase its service business revenue by 88.7% in 2021 compared to that of the previous year and further improve its service quality and efficiency.

### 4.3. Robustness test

Typically, the robustness test of fsQCA involves adjusting the consistency level threshold, adjusting the calibration anchor point, and performing asymmetric causality analysis. First, referring to existing studies, the adequacy consistency level threshold is adjusted from 0.8 to 0.85, the PRI consistency level threshold is increased to 0.7, and the other treatments remain unchanged. Second, on this basis, the 90%, 50% and 10% quantile values of the outcome variables and condition variables are used as the determination points for “full affiliation”, “intersection” and “full nonaffiliation”, respectively. The test results are presented in Table 6. The overall consistency of the solutions remains unchanged under the two test methods; however, the coverage of the solutions slightly decreases. Increasing the adequacy and PRI consistency level thresholds reduces the number of truth table rows and cases included in the minimisation analysis, which in turn leads to a reduction in grouping. The core variables in Columns A and B of Table 6 are identical to those in Groups A1 and A2 of Table 5. Columns C and D in Table 6 are identical to the core variables in Groups B and C in Table 5, respectively. An asymmetric causality test was then conducted. According to this principle, Hypothesis X1 + X2 + ... + Xn → Y does not necessarily follow that ~X1 + –X2 + ... + ~Xn→~Y. By comparing the results of the non-servicing transformation group path and the results of the group path of the servicing transformation, it is found that they are not the same (chart omitted). In summary, after analysing the three tests, the group path results of the servitisation transformation of the manufacturing industry did not change significantly, indicating that the results of the study are highly robust.

**Table 6:** Results of the robustness test.

## 5. Conclusions and implications

### 5.1. Conclusion and discussion

Given the current increase in production costs in China’s sporting goods manufacturing industry, serious homogenisation of products, low-priced competition, and other issues, there is an urgent need to break through the ‘low-end lock’ in the sporting goods industry and climb to the high end of the industry’s value chain. Servitisation, transformation, and upgrading are important ways to advance the structure of China’s sporting goods manufacturing industry. However, the core conditions affecting the transformation of servitisation in China’s sporting goods manufacturing industry and its complex interaction mechanisms have not been deeply explored in existing studies. Therefore, this study considers 33 sporting goods manufacturing enterprises listed on the Shanghai and Shenzhen A-shares and New Third Board as research objects and performs a condition grouping analysis using the TOE framework and the fsQCA method. It explores the driving paths of the technological, organisational, and environmental dimensions affecting the performance of China’s sporting goods manufacturing industry’s servitisation transformation. The following conclusions were drawn:

(1) Technology and environmental conditions alone do not constitute the necessary conditions for transforming the servitisation performance of sporting goods manufacturing firms and require coordination between factors under multidimensionality, a conclusion that is still valid after the results of the robustness test. The degree of digital transformation as a technology dimension condition appears to be a core condition in both Groups C and D, reflecting the important role of digital transformation in the servitisation of sporting goods manufacturing firms.
(2) The conclusion that organisational dimension conditions alone constitute the necessary conditions for the performance of sporting goods manufacturing firms in service transformation still holds after the results of the robustness test. This study further confirms that improving the internal environment of firms through staff knowledge structure and organisational redundancy alone, without relying on external environmental factors, can also enhance the level of servitisation in the sporting goods manufacturing industry in China.
(3) Three types of conditional configuration paths constitute the driving path for service-oriented transformation and upgrading of sporting goods manufacturing enterprises in China. These include “talent–resource driven”, which emphasises staff knowledge structure and organisational redundancy; and “technology–organisation driven” and “technology–talent driven”, which emphasise digital technology, staff knowledge structure and organisational redundancy; and “technology–talent driven”, which emphasises digital technology and staff knowledge structure and organisational redundancy. Technology–talent driven” emphasises digital technology and employee knowledge. Among them, the “talent–resource driven” path plays the most important role. This enriches qualitative research on the servitisation of China’s sporting goods manufacturing industry from a quantitative perspective [9,25,26] and provides more cases of enterprise servitisation to provide new ideas for subsequent researchers.

### 5.2. Implications

The improved servitisation transformation of China’s sporting goods manufacturing industry is the result of the combined effect of multiple factors. In which the organisational dimension is more important than the technical and environmental dimensions, and the reasonable combination of the seven influencing factors on the transformation performance of the servitisation of sporting goods manufacturing enterprises is “the same way”, i.e., the three types of grouping paths have a significant impact on the performance of the servitisation transformation of sporting goods manufacturing enterprises, which is the same as the other conditions. That is, the impact of the three types of group paths on the development of the servitisation transformation of sporting goods manufacturing enterprises is consistent. Therefore, enterprises should combine their own characteristics and development concepts, identify and select the core and non-core conditions in each grouping path, and choose suitable conditions to develop a servitisation business. Moreover, the three factors influencing digital transformation, staff knowledge structure, and organisational redundancy in the three types of group paths of the servitisation transformation of the sporting goods manufacturing industry play a major role in promoting each path. Thus, China’s sporting goods manufacturing enterprises must pay great attention to the development of these three factors in the servitisation transformation process. As a core requisite, an staff knowledge structure appears in each of the grouping paths, with organisational redundancy and the degree of digital transformation in the second and third places in terms of the number of occurrences.

Therefore, the focus of the service-oriented transformation and upgrading of China’s sporting goods manufacturing enterprises is as follows: (1) Further strengthening the introduction and cultivation of highly educated and skilled compound talent, simultaneously providing a stable platform and financial support for innovative activities of elite employees and playing a role in attacking the innovation of service products and driving the enhancement of the overall innovation ability of enterprise employees. (2) Enhancing the redundant resource creation ability of business operators through diversified investment and optimal resource allocation, improving the knowledge sharing platform, optimising cash flow management, and enhancing the redundant calling ability of managers by establishing efficient information systems, optimised supply chain management, and rapid decision-making mechanisms. (3) Actively build an intelligent digital platform based on cloud computing and microservice architecture to achieve full-process data intercommunication. Focusing on introducing cutting-edge technologies such as industrial Internet of Things, artificial intelligence, and blockchain, and achieve seamless integration with existing systems through standardized API interfaces to eliminate information silos and create a data-driven intelligent decision-making mechanism. In terms of supply chain optimization, intelligent warehouse management systems and supplier collaboration platforms are deployed to reduce inventory costs. In response to personalized demands, a customer profiling system is built to achieve a transformation from mass production to mass customization, comprehensively enhancing customer experience and product added value.

### 5.3. Limitations and future research

This study examines the factors influencing the servitisation performance of China’s sporting goods manufacturing industry via the TOE framework and fsQCA methodology, using a sample of 33 listed companies from the Shanghai and Shenzhen A-share markets and the New Third Board. This study presents robust conclusions, however, this study has several limitations. (1) As the conceptual scope of sporting goods manufacturing enterprises in China has not been precisely defined, there may be some omissions of listed enterprises in this study’s sample. In addition, because mandatory disclosure of corporate financial data is not required for unlisted companies in China, the financial data of unlisted small and medium-sized sporting goods manufacturing companies are unavailable, making it impossible to include them in the research sample. (2) Due to the limitations of the research methodology and software, fsQCA can only use one year of cross-sectional data for analysis, which may impact the precision of the results. Qualitative comparative analysis offers the possibility of incorporating time into combinatorial analyses such as calibrating ensemble affiliation in the temporal dimension or dynamically linking a series of causal models. However, specific technical applications need to be developed.

Therefore, future studies should focus on the following aspects. (1) Using a questionnaire survey and field research, financial data of small and medium-sized sporting goods manufacturing enterprises in different regions of China to explore the impact of different factors on the servitisation transformation of sporting goods manufacturing enterprises of different sizes to conduct a more comprehensive and accurate analysis of the servitisation transformation of these enterprises. (2) Further improvement of the research methodology, such as developing a dynamic QCA methodology using R language software to realise the organic linkage between panel data and the QCA methodology, measuring three dimensions—between groups, within groups, and aggregated—and further expanding the measurement dimensions of traditional QCA to arrive at more accurate conclusions through a larger sample size.

## Supporting information

S1 File Data. (XLSX)
